# Selection and stability validation of reference gene candidates for transcriptional analysis in *Rousettus aegyptiacus*

**DOI:** 10.1038/s41598-021-01260-z

**Published:** 2021-11-04

**Authors:** Virginia Friedrichs, Anne Balkema-Buschmann, Anca Dorhoi, Gang Pei

**Affiliations:** 1 Institute of Immunology, Friedrich-Loeffler-Institut, Greifswald-Insel Riems, Germany; 2 Institute of Novel and Emerging Infectious Diseases, Friedrich-Loeffler-Institut, Greifswald-Insel Riems, Germany; 3 Faculty of Mathematics and Natural Sciences, University of Greifswald, Greifswald, Germany

**Keywords:** Immunology, Molecular biology

## Abstract

Bats are the only mammals capable of powered flight and their body temperature can reach up to 42 °C during flight. Additionally, bats display robust type I IFN interferon (IFN-I) responses and some species constitutively express IFN-α. Reference genes with stable expression under temperature oscillations and IFN-I release are therefore critical for normalization of quantitative reverse-transcription polymerase chain reaction (qRT-PCR) data in bats. The expression stability of reference genes in *Rousettus aegyptiacus* remains elusive, although this species is frequently used in the infection research. We selected *ACTB*, *EEF1A1*, *GAPDH* and *PGK1* as candidate reference genes and evaluated their expression stability in various tissues and cells from this model bat species upon IFN-I treatment at 35 °C, 37 °C and 40 °C by qRT-PCR. We employed two statistical algorithms, BestKeeper and NormFinder, and found that *EEF1A1* exhibited the highest expression stability under all tested conditions. *ACTB* and *GAPDH* displayed unstable expression upon temperature change and IFN-I treatment, respectively. By normalizing to *EEF1A1*, we uncovered that *GAPDH* expression was significantly induced by IFN-I in *R. aegyptiacus*. Our study identifies *EEF1A1* as the most suitable reference gene for qRT-PCR studies upon temperature changes and IFN-I treatment and unveils the induction of *GAPDH* expression by IFN-I in *R. aegyptiacus.* These findings are pertinent to other bat species and may be relevant for non-volant mammals that show physiological fluctuations of core body temperature.

## Introduction

Bats are increasingly recognized as reservoir hosts of highly-virulent pathogens, such as Filoviruses, Lyssaviruses, Paramyxoviruses and Coronaviruses, including severe acute respiratory syndrome coronavirus 2 (SARS-CoV-2), which causes the current global pandemic^1^. Egyptian fruit bats, *Rousettus aegyptiacus*, have been identified as putative reservoir hosts of Marburg virus^2^, Kasokero virus^3^ and Sosuga virus^4^, and were shown to be susceptible to experimental challenge with SARS-CoV-2^5^ and Rift valley fever phlebovirus^6^. These viruses may cause severe diseases with high mortality rates in humans. However, bats show minimal and often even no clinical manifestation upon natural infection. Under experimental conditions, *Rousettus* bats infected with high doses of Ebola^7^, SARS-CoV^8^ or SARS‐CoV‐2^5^ only support transient viral replication and display limited pathology. Several hypotheses could explain the reservoir potential of bats. As the only mammals capable of powered flight, their body temperature can reach 42 °C during flight^9^. Hence, ‘flight-as-fever’ has been postulated as a unique mechanism conferring effective immune defence^6^. Further, the black flying fox, *Pteropus alecto*, constitutively expresses IFN-α^10^, and gene loci of type I interferons (IFN-I) in *R. aegyptiacus*, are markedly expanded^11^, indicating a potential contribution of boosted IFN-I signalling to antiviral immunity in bats. In addition to the enhanced antiviral immunity, bats can suppress excessive inflammation which may explain their lack of symptoms during viral infection^1,12^. For example, *TNFα* expression in *Eptesicus fuscus* cells is abolished upon poly(I:C) stimulation^13^, NLRP3-mediated inflammasome activation is impaired in *P. alecto*^14^ and *Myotis davidii*^12^, and *R. aegyptiacus* does not upregulate pro-inflammatory genes (*CCL8*, *FAS* and *IL6*) upon Marburg virus infection^15^. Enhanced antiviral immunity along with reduced inflammation likely explains the ability of bats to harbour high-impact pathogens in absence of clinical disease. Confirmation of these findings in distinct bat species as well as elucidation of novel immune mechanisms contributing to the reservoir potential of bats require accurate monitoring of their immune responses.

The knowledge about the exceptional immune system of bats has significantly advanced during the past decade^16^. However, experimental tools to systematically investigate bat immune responses, such as species-specific or cross-reactive antibodies, are largely missing^17–19^. Accordingly, investigations on host immunity heavily rely on gene transcription profiling by qRT-PCR. This method is sensitive, specific, highly reproducible and accurate^20,21^. A critical step in qRT-PCR setup is selection of several stable reference genes. The inclusion of such reference genes is crucial for gene expression normalization and subsequent data interpretation. The ideal reference genes should maintain stable expression levels across diverse tissues and cell types as well as under different experimental conditions^22^. Considering that bats display unique physiological features, notably oscillating metabolic rates and core body temperature depending on flying and roosting phases^23–26^, the expression stability of reference genes must be evaluated in context of these physiologically relevant conditions. Multiple reference genes, including glyceraldehyde-3-phosphate dehydrogenase (*GAPDH*), actin-beta (*ACTB*), small nuclear ribonucleoprotein Sm D3 (*SNRPD3*) and 18S ribosomal RNA (*18S rRNA*) have been employed in gene expression studies of *P. alecto*, *E. fuscus*, *M. davidii* and other bats^13,14,27–29^. However, a comprehensive analysis of reference genes, particularly their expression stability under oscillating temperatures, has not been performed in bats, including the model bat *R. aegyptiacus*.

Here, we provide a first in-depth validation of four reference gene candidates, including *ACTB*, *GAPDH*, eukaryotic translation elongation factor 1 alpha 1 (*EEF1A1*) and phosphoglycerate kinase 1 (*PGK1*) for *R. aegyptiacus*. Our findings support *EEF1A1* as an appropriate reference gene for normalization of qRT-PCR data in the Egyptian fruit bat and call for caution when using other candidate genes, i.e. *GAPDH* and *ACTB*, due to their instability under specific conditions.

## Results

### Performance of PCR primers targeting reference gene candidates

To evaluate the performance of the qRT-PCR assay, we first examined the specificity of the primer pairs with melting curve analysis, agarose gel electrophoresis and sequencing. Melting curve analysis revealed single peaks for all primer pairs (Fig. S1). Agarose gel electrophoresis further demonstrated single bands for all the PCR products with the predicted sizes, indicating high specificity of all primer pairs (Fig. S1). PCR products were sequenced and the specificity of the primers was confirmed (Fig. S2). To evaluate the amplification efficiency, standard curves with ten-fold dilution steps were generated (Fig. S3) and subsequently the linear dynamic range (LDR) and precision of each primer pair were assessed following the MIQE guideline^30^. Amplification efficiencies of all tested primers met the validation criteria, notably 100.51% for *ACTB*, 99.53% for *EEF1A1*, 106.52% for *GAPDH* and 106.44% for *PGK1* (Table 1). The correlation coefficient (R^2^) of all candidates was above 0.99, suggesting excellent linearity of the standard curves. The LDR values of all primers were in the range of 3 to 300,000 copies and precision values varied from 0.31 (*EEF1A1*) to 1.27 (*ACTB*) (Table 1). Thus, all the primers for reference gene candidates demonstrated satisfactory specificity and efficiency in qRT-PCR.

**Table 1:** Summary of the performance of primers employed in this study.

Gene symbol	Efficiency [%]	Slope	LDR (copies)	Precision (SD of intra-assays)	Correlation coefficients (R^2^)
*ACTB*	100.51	−3.3097	3–300,000	1.27	0.9999
*EEF1A1*	99.53	−3.3333	3–300,000	0.31	0.9969
*GAPDH*	106.52	−3.175	3–300,000	0.66	0.9991
*PGK1*	106.44	−3.1767	3–300,000	0.32	0.9976

### Expression profile of reference gene candidates in various tissues from *R. aegyptiacus*

**Figure 1:**
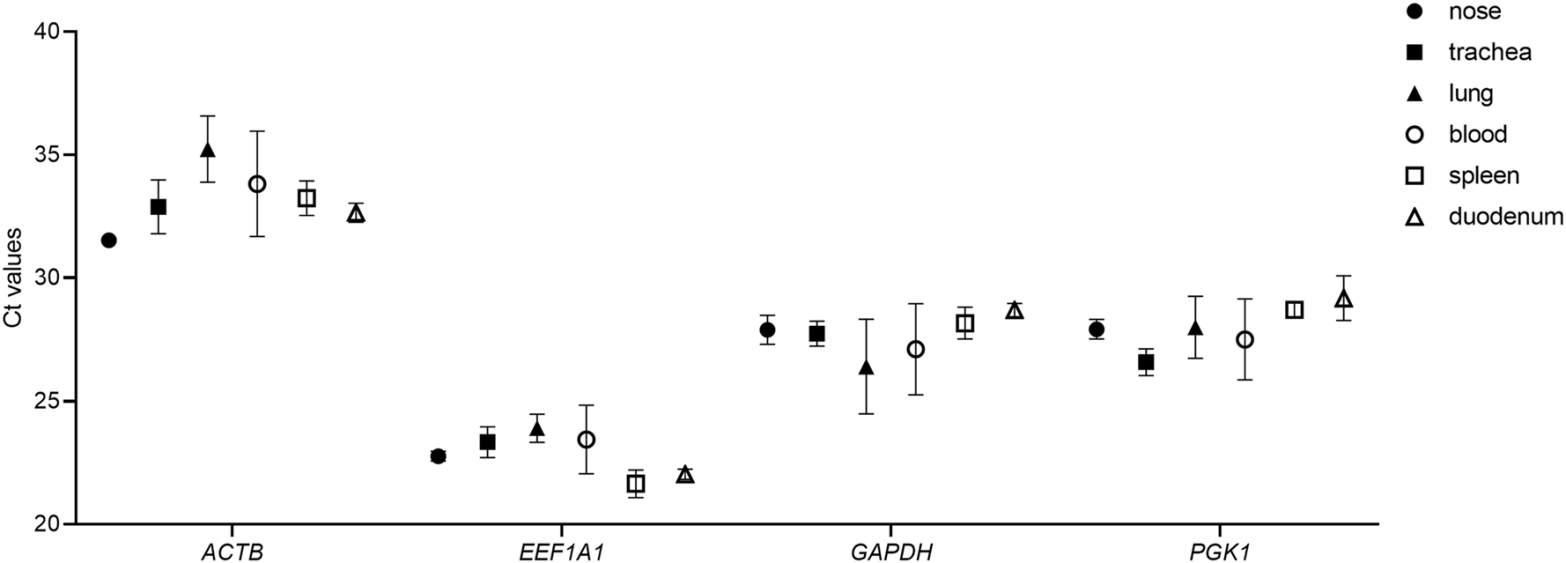
Ct values of selected reference genes in various tissues from *Rousettus aegyptiacus*. RNA was extracted from nose, trachea, lung, blood, spleen and duodenum from 12 animals. Expression of reference gene candidates were determined via qRT-PCR. Data show mean ± SD from 3 independent experiments.

To investigate the expression of reference gene candidates in tissues from *R. aegyptiacus*, qRT-PCR was performed with pooled cDNA from nose (nasal epithelium), trachea, lung, blood, spleen and duodenum. Threshold cycle (Ct) values were employed to determine the expression levels of the candidate reference genes. Their expression levels varied and *EEF1A1* displayed the highest expression levels across different tissues as indicated by the lowest Ct values. The overall Ct values of *EEF1A1*, *ACTB*, *GAPDH* and *PGK1* were 23 ± 1.5, 33 ± 3, 27 ± 2 and 27 ± 1.5, respectively (Fig. 1). *EEF1A1* and *PGK1* showed the lowest variability in Ct values, suggesting that these two genes display the most stable expression across diverse tissues from *R. aegyptiacus*.

### Expression of candidate reference genes in primary bat fibroblasts upon IFN-I stimulation and incubation at various temperatures

**Figure 2:**
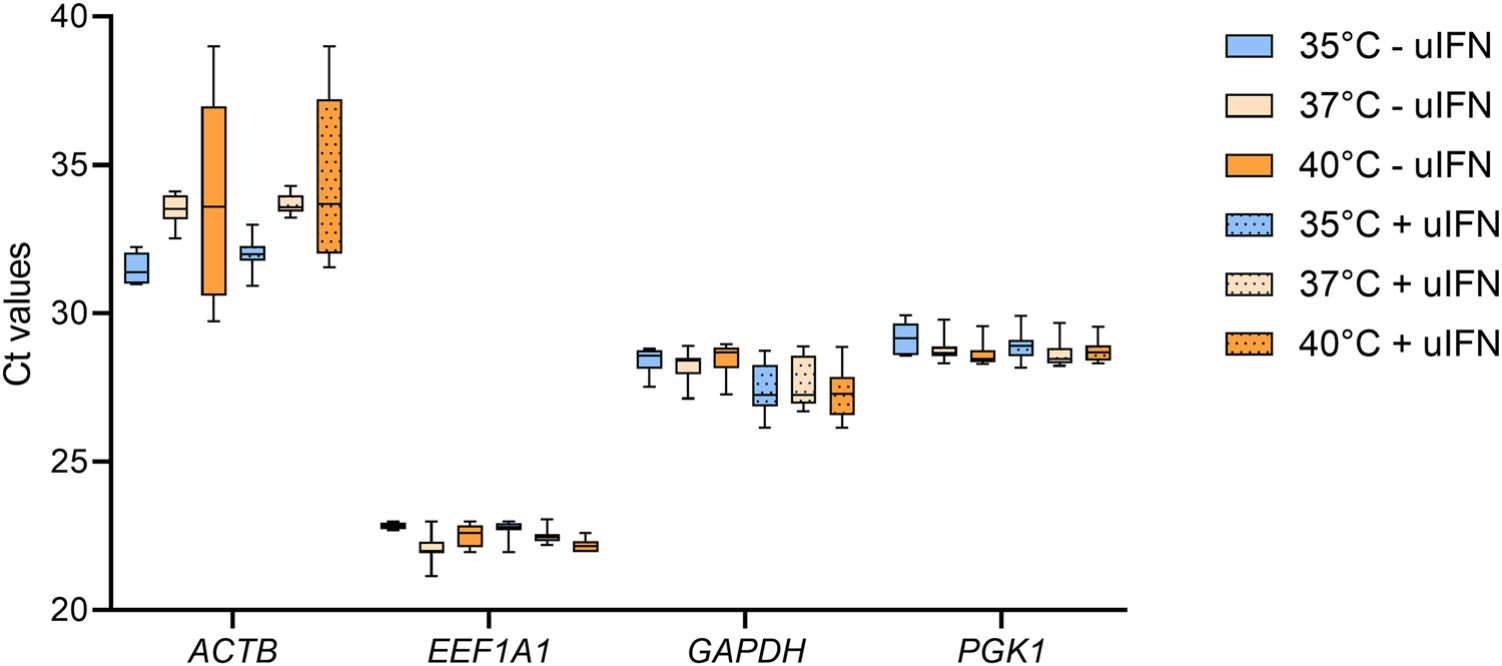
Expression stability of candidate reference genes under different conditions. Bat primary fibroblasts were incubated at 35 °C, 37 °C or 40 °C in the presence or absence of 1,000 U/ml universal type I interferon (uIFN). Gene expression levels of *ACTB*, *EEF1A1*, *GAPDH* and *PGK1* were determined by qRT-PCR. Data show mean ± SD from 3 independent experiments.

To investigate the expression stability of candidate reference genes, bat primary fibroblasts were incubated at 35 °C, 37 °C or 40 °C in the presence or absence of universal type I interferon (uIFN) for 4 h. We employed 35 °C, 37 °C and 40 °C to mimic the physiological daily oscillation of body temperature in *R. aegyptiacus* ^9^. The expression levels of all candidate reference genes under these conditions were assessed by qRT-PCR. *ACTB* showed a broad variation in Ct values, ranging from 29.7 to 39.01 at 40 °C. The Ct values of *ACTB* were lower at 35 °C, 31.5 ± 0.7 compared to 33.5 ± 1 at 37 °C, suggesting an unstable expression of *ACTB* upon temperature changes (Fig. 2). The expression levels of *EEF1A1*, *GAPDH* or *PGK1* were comparable at 35 °C, 37 °C and 40 °C, demonstrating stable expression of these potential reference genes under temperature oscillations. Since uIFN activates IFN-I pathway in bats^31^, we employed this cytokine to further investigate the expression of the candidate reference genes upon IFN-I stimulation. The mean Ct values of *GAPDH* decreased upon IFN-I stimulation at 35 °C, 37 °C and 40 °C, indicating increased expression of *GAPDH* by IFN-I. The Ct values of *EEF1A1* and *PGK1* remained unchanged following stimulation with IFN-I at 35 °C, 37 °C or 40 °C, suggesting their stable expression under these experimental conditions (Fig. 2). These findings indicate that expression stabilities of *ACTB* and *GAPDH* are impaired by temperature oscillations and IFN-I, respectively.

### Expression stability analysis of reference gene candidates under different conditions

To quantitatively evaluate the expression stability of candidate reference genes, qPCR results were analysed with the statistical algorithms BestKeeper and NormFinder. BestKeeper enables pairwise correlation, regression analysis^32,33^ and calculations of standard deviation (SD) of all Ct values [SD (± Ct)] as well as of the standard deviation of absolute regulation coefficients [SD (± x-fold)]. Both parameters are indicators of expression variability. A suitable reference gene should display values of < 1 for SD (± Ct), < 2 for SD (± x-fold), and have a coefficient of correlation (*r*) close to 1 ^34^. According to this algorithm, the [SD (± Ct)] values for *ACTB*, *EEF1A1*, *GAPDH* and *PGK1* were 1.40, 0.34, 0.76 and 0.36, along with their corresponding *r*-values of 0.52, 0.96, 0.78 and 0.88. Thus, expression stability for candidate reference genes ranks as following: *EEF1A1*, *PGK1*, *GAPDH*, and *ACTB* (Table 2).

**Table 2:** Stability analysis of reference gene candidates based on pairwise correlations by BestKeeper.

Parameter	Reference gene			
	ACTB	EEF1A1	GAPDH	PGK1
geo Mean [CP]	33.19	22.44	27.86	28.78
ar Mean [CP]	33.25	22.44	27.87	28.79
min [CP]	29.74	21.14	26.14	28.17
max [CP]	39.00	23.07	28.96	29.93
std dev [± CP]	1.40	0.34	0.76	0.36
CV [% CP]	4.22	1.53	2.73	1.25
min [x-fold]	−10.97	−2.46	−3.29	−1.53
max [x-fold]	55.98	1.55	2.15	2.22
std dev [± x-fold]	2.64	1.27	1.69	1.28
coeff. of corr. [r]	0.52	0.96	0.78	0.88
p-value	0.02	0.00	0.01	0.00

NormFinder was employed as the second algorithm to determine gene stability, since it allows evaluation of the overall stability as well as the individual stability for each condition^35^. The most stable reference genes display stability values close to 0 according to this algorithm. Based on this method, we calculated the overall and individual stability values. The total stability values for *ACTB*, *EEF1A1*, *GAPDH* and *PGK1* were 0.097, 0.013, 0.05 and 0.015, respectively, suggesting *EEF1A1* as the most stable reference gene under all tested conditions. *EEF1A1* also displayed the highest expression stability under high temperature or upon IFN-I treatment (Table 3). Altogether, both algorithms suggest that *EEF1A1* is the reference gene with the highest expression stability under the selected conditions, followed by *PGK1*, *GAPDH*, whereas *ACTB* has the lowest stability.

**Table 3:** Stability analysis of reference gene candidates based on estimated expression variations by NormFinder.

	Stability in various conditions		
	Total	uIFN	Temperature
EEF1A1	0.013	0.015	0.014
PGK1	0.015	0.017	0.016
GAPDH	0.050	0.069	0.049
ACTB	0.097	0.085	0.170

### *GAPDH* expression is induced by IFN-I in *R. aegyptiacus* cells

**Figure 3:**
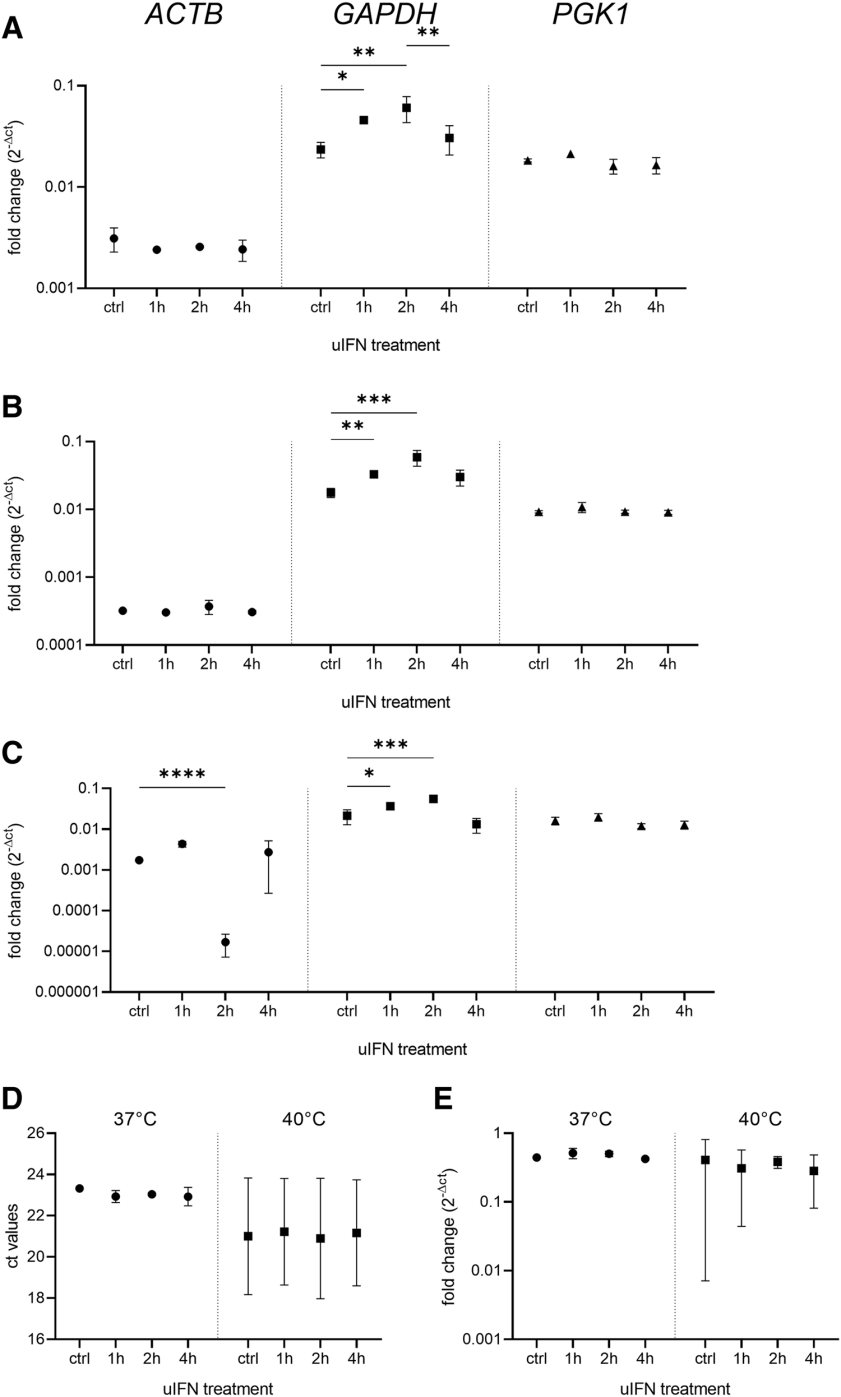
*GAPDH* expression is induced by IFN-I in *R. aegyptiacus*. Relative gene expression of *ACTB*, *GAPDH* and *PGK1* normalized to *EEF1A1* upon uIFN treatment at 35 °C (**A**), 37 °C (**B**) or 40 °C (**C**). (**D, E**) Ct values of human *ACTB* and *GAPDH* (**D**) and relative expression of human *GAPDH* normalized to *ACTB* (**E**) in human fibroblasts upon uIFN treatment and incubation at 37 °C or 40 °C. Bat (**A, B, C**) or human (**D, E**) primary fibroblasts were stimulated with PBS (ctrl) or 1000U/ml universal type I interferon (uIFN) for indicated time at 35 °C, 37 °C or 40 °C. Gene expression levels of *ACTB*, *GAPDH* and *PGK1* were determined with qRT-PCR. Data show mean ± SD from 3 independent experiments. The statistical significance was calculated using one-way ANOVA with Holm-Šidák’s post-hoc test. (*) *P* ≤ 0.05, (**) *P* ≤ 0.01, (***) *P* ≤ 0.001, (****) *P* ≤ 0.0001.

To evaluate the relative expression levels of *ACTB*, *GAPDH* and *PGK1* under different conditions, we normalized each candidate to the most stable reference gene, *EEF1A1* (Fig. 3A–C). The relative expression levels of *PGK1* remained stable at all temperatures evaluated, irrespective of the IFN-I treatment. *ACTB* expression was also stable at 35 °C and 37 °C in the presence of IFN-I. However, the expression level was significantly reduced following a 2 h IFN-I stimulation at 40 °C (Fig. 3C). Intriguingly, the expression of *GAPDH* was significantly increased in cells treated with IFN-I at all selected temperatures, suggesting that the induction of *GAPDH* by IFN-I is temperature independent in *R. aegyptiacus* (Fig. 3A–C). To investigate whether *GAPDH* induction by IFN-I is specific to *R. aegyptiacus*, we stimulated human fibroblasts with IFN-I at 37 °C and 40 °C (Fig. 3D, E). Both human *GAPDH* and *ACTB* have been previously used as reference genes in several qRT-PCR studies^36,37^. Indeed, both genes displayed good expression stability at 37 °C, yet the variation of Ct values at 40 °C argued against stability of human *GAPDH* and *ACTB* at 40 °C. The relative expression of human *GAPDH* normalized against *ACTB* remained unchanged upon IFN-I treatment either at 37 °C or 40 °C, revealing that human *GAPDH* expression in fibroblasts is not modulated by IFN-I (Fig. 3D, E). Overall, we conclude that IFN-I triggers *GAPDH* expression specifically in *R. aegyptiacus*.

## Discussion

Accurate gene transcription measurements require selection of reference genes that maintain high stability under various experimental conditions. In this study, we selected *ACTB*, *GAPDH*, *EEF1A1* and *PGK1* as reference gene candidates due to their wide applications in other species^14,32,36,39,42–48^ and evaluated the suitability of these candidates in *R. aegyptiacus* under the physiological relevant conditions, notably temperature oscillation and IFN-I stimulation. By employing BestKeeper and NormFinder, we calculated the stability and validated *EEF1A1* as the most stable reference gene in *R. aegyptiacus* under conditions relevant for the biology of this species^36,38–41^. By normalizing the qRT-PCR data to *EEF1A1* we observed that expression of *GAPDH* was significantly induced by IFN-I, suggesting unsuitableness of using this commonly used reference gene in *R. aegyptiacus*. Consistent with our results, many reports have demonstrated that the expression of *GAPDH* and *ACTB* is unstable in various tissues or cells from mice and humans and upon certain stimulations, such as IL-2 and hypoxia ^37,49–52^. Whether *GAPDH* expression is induced by IFN-I, and whether *GAPDH* and *ACTB* are suitable reference genes in other bat species, require further investigations.

Our finding that the expression of *GAPDH*, one of the key enzymes in glycolysis, is induced by IFN-I in *R. aegyptiacus* may have implications for the immunometabolism of bats. It is well established that metabolic reprogramming in cells controls their immune responses^53,54^. Glycolysis is commonly utilized by various immune cells to enable prompt responses to infections. In bone marrow derived macrophages, aerobic glycolysis promotes IL-1β production upon LPS stimulation or *Bordetella pertussis* infection ^55^. In CD4^+^ and CD8^+^ T-cells^56–60^, as well as in NK cells^61,62^, glycolysis is required for their effector functions, such as IFN-γ production and cytotoxicity. In human plasmacytoid dendritic cells (pDCs) and monocyte-derived DCs (moDC), glycolysis promotes IFN-I production upon TLR9 or RIG-I activation, respectively^63^. Further, IFN-I induces a metabolic shift towards glycolysis, contributing to the antiviral activity in fibroblasts and antigen presentation in DCs^64,65^. On the other hand, lactate, the end metabolite of glycolysis, directly binds to mitochondrial antiviral-signaling protein (MAVS), and consequently inhibits its activation and IFN-I production^66^. Thus, outcomes of such metabolic shift vary in diverse cells or under different stimulations. The nectarivore bat *Glossophaga soricina* employs high rates of glycolysis to generate ATP during flight^67^. As a fruit bat, *R. aegyptiacus* could also directly utilize dietary sugars to fuel both roosting and flight metabolism^68^. Whether induction of *GAPDH* expression in this species impacts on metabolic reprogramming towards glycolysis, needs to be clarified. Moreover, whether and how such metabolic shifts affect IFN-I signalling or other immune pathways in *R. aegyptiacus* remains to be uncovered.

In addition to its roles in glycolysis, *GAPDH* also modulates cell death, RNA export and cytoskeleton dynamics^69^. Upon serum deprivation and DNA damage, *GAPDH* translocates to the mitochondria and interacts with voltage-dependent anion channel (VDAC), leading to apoptosis^70^. It can also bind to the 3' untranslated region of *TNFα* mRNA and represses TNFα expression in human monocytes and macrophages^71^. Hence, *GAPDH* upregulation by IFN-I may contribute to apoptosis induction and TNFα repression in *R. aegyptiacus*, which could represent novel mechanisms for the prevention of excessive inflammation during viral infections.

Overall, our study provides an extensive analysis of reference genes and identifies *EEF1A1* as the most stable reference gene in *R. aegyptiacus* under temperature changes and IFN-I stimulation, which allows us to perform accurate gene transcription studies in this species. Our findings also open new investigation avenues by showing that *GAPDH* is regulated by IFN-I which has a broad relevance in context of immunometabolism.

## Material and methods

### Selection of reference gene candidates and design of primer pairs

Reference gene candidates (*ACTB*, *EEF1A1*, *GAPDH* and *PGK1*) for *R. aegyptiacus* gene expression studies were selected based on their utilization in other bat species^13,14,17^. Primers against these reference genes were designed using the PrimerQuest tool (Integrated DNA Technologies, Inc.). The criteria for primer design were as follows: primer lengths around 17–30 bp, GC content of 40–55%, optimal melting temperature at 62 °C, and amplicon lengths within a range of 100–250 bp. Derived primer pairs were evaluated using the OligoAnalyzer tool to exclude primers with hairpin structures and homo- and/or heterodimer formation (Integrated DNA Technologies, Inc.). Primer sequences were also blasted using the NCBI BLAST tool to ensure their specificity for *R. aegyptiacus*. The primer pairs meeting all criteria were selected for further experiments. The characteristics of these primers are shown in Table 4.

**Table 4:** Summary of selected reference gene candidates.

Gene symbol	Gene name	Gene ID	Primer sequence [5′–3′]	Amplicon size [bp]
*ACTB*	actin beta	107515934	F-GCCTTGGTCGTGGATAATG R-GGGATACTTCAGGGTCAGGATA	193
*EEF1A1*	eukaryotic translation elongation factor 1 alpha 1	107509282	F-GTATGCCTGGGTCTTGGATAAA R-GCCTGTGATGTGCCTGTAA	162
*GAPDH*	glyceraldehyde-3-phosphate dehydrogenase	107519804	F-CAAGTTCAAAGGCACAGTCAAG R-TATTCAGCACCAGCATCACC	120
*PGK1*	phosphoglycerate kinase 1	107503843	F-GATTACCTTGCCTGTTGACTTTG R-GACAGCCTCAGCATACTTCTT	148

### Cells, tissues and stimulation experiments

Bat fibroblasts were derived from the lung of a female *R. aegyptiacus* bat from the *R. aegyptiacus* breeding colony at the Friedrich-Loeffler-Institut. Sampling was performed in accordance with current European and National Animal Welfare regulations, after ethical review and approval by the authority of the Federal State of Mecklenburg-Western Pomerania, Germany (file number 7221.3–2-042/17) and the experiments were carried out according to ARRIVE guidelines (https://arriveguidelines.org). The lung tissue was dissected into small pieces and digested with trypsin overnight at 4 °C. Dissociated cells were seeded in cell culture dishes in DMEM medium (DMEM high glucose medium, 10% fetal bovine serum (FBS), 2 mM glutamine and 100U/mL of Penicillin–Streptomycin) for 2 h, and only adherent cells were propagated. Confirmation of bat fibroblast identity was carried out by examining fibroblast activation protein (FAP) expression via PCR (data not shown).

Tissue samples from 12 individual bats were obtained from an animal experiment published before^76^. For IFN-I stimulation, bat fibroblasts and human dermal fibroblasts (#C0045C, Thermo Fisher Scientific) were incubated with 1000U/ml universal IFN-I (uIFN) (#11200-1, PBL Assay Science) for 1 h, 2 h and 4 h at either 35 °C, 37 °C or 40 °C, respectively.

### RNA extraction and cDNA synthesis

Cells were lysed in homemade Trizol solution and RNA was extracted as published before^77^. Purified RNA was quantified using NanoDrop 2000c spectrophotometer (#ND-2000c, Thermo Fisher Scientific) and 800 ng RNA were subsequently utilized for cDNA synthesis with the LunaScript RT SuperMix Kit (#E3010L, New England BioLabs).

### qRT-PCR

qRT-PCR reactions were carried out with EvaGreen Fluorescent DNA stain (# PCR-379, Jena Bioscience), ROX as an internal reference dye (#PCR-351, Jena Bioscience) and GoTaq Polymerase (#M3001, Promega) according to manufacturers’ instructions. The reaction setup was as follows: 95 °C for 2 min; (95 °C for 30 s, 62 °C for 30 s, 72 °C for 1 min) for 40 cycles; 72 °C for 10 min and infinite hold at 4 °C. Unless stated otherwise, each qPCR reaction was performed with 100 ng cDNA. To minimize pipetting errors, the template was diluted and 5 µl were used for each qRT-PCR reaction. Each primer pair was added separately into different wells. Measurements were performed with the QuantStudio 6 Flex real-time PCR system (#4485691, Applied Biosystems). Melting curves were performed within the temperature range from 60.16 °C to 94.885 °C in steps of 0.193 °C, respectively.

### Establishment of standard curves and examination of amplification efficiency via qRT-PCR

Standard curves of all reference genes in the qRT-PCR reaction were generated with copy numbers from 300,000 to 3 copies in ten-fold dilution steps. To achieve accurate copy numbers, amplicon sizes of each reference gene were used to calculate the specific weight of each amplicon^78^, resulting in 2.12 × 10^–19^ g for *ACTB*, 1.78 × 10^–19^ g for *EEF1A1*, 1.32 × 10^–19^ g for *GAPDH* and 1.62 × 10^–19^ g for *PGK1*. The amplification efficiency of all primer pairs was subsequently determined with the slope of the standard curve according to the equation 10^–1^^/slope^-1. The amplification efficiency of favourable primers ranges between 90–110%. Linear dynamic range (LDR) is described as the highest to the lowest quantifiable copy numbers from standard curves. LDR should cover at least 3 orders of magnitude, ideally 5–6 orders. Precision refers to intra-assay variation and is defined as standard deviation (SD) of technical replicates^30^.

### Amplicon purification and sequencing

Amplicons of all candidate reference genes were visualized in 1.5% agarose gels and bands were cut and purified using the QIAquick gel extraction kit (#28506, Qiagen). Purified amplicons were subsequently sequenced using the Eurofins tubeseq platform.

### Stability analyses of reference gene candidates

To investigate the expression stability of the four reference gene candidates, two statistical algorithms were used: BestKeeper^33^ and NormFinder^35^. The highest stability was defined as the lowest variation of expression levels under all the selected experimental conditions^33,35^. In brief, for BestKeeper, raw Ct values without any normalization are subjected to the calculation and the parameters of interest are SD (std dev [± CP]) and the Pearson coefficient of correlation (r). NormFinder utilizes normalized Ct values (2^−ΔCt^) to provide a direct readout for the estimated expression variation and can separately calculate the stability under each condition (IFN-I treatment, temperature) or total stability.

### Statistical analysis

Statistical analysis was performed with GraphPad Prism 8 (GraphPad Software Inc., USA). To determine statistical significance among investigated groups, one-way analysis of variance (ANOVA) with Holm-Šidák’s post-hoc test was performed. A *P* value of < 0.05 was considered to be significant.

## Supplementary Information

Supplementary Information 1.

Supplementary Information 2.

## References

[1] Irving AT, Ahn M, Goh G, Anderson DE, Wang L-F (2021). Lessons from the host defences of bats, a unique viral reservoir. Nature.

[2] Amman BR (2015). Oral shedding of Marburg virus in experimentally infected Egyptian fruit bats (*Rousettus aegyptiacus*). J. Wildl. Dis..

[3] Kalunda M (1986). Kasokero virus: A new human pathogen from bats (*Rousettus aegyptiacus*) in Uganda. Am. J. Trop. Med. Hyg..

[4] Amman BR (2015). A recently discovered pathogenic paramyxovirus, Sosuga virus, is present in *Rousettus aegyptiacus* fruit bats at multiple locations in Uganda. J. Wildl. Dis..

[5] Schlottau K (2020). SARS-CoV-2 in fruit bats, ferrets, pigs, and chickens: an experimental transmission study. Lancet. Microbe.

[6] Balkema-Buschmann A (2018). Productive propagation of rift valley fever phlebovirus vaccine strain MP-12 in *Rousettus aegyptiacus* fruit bats. Viruses.

[7] Paweska JT (2016). Experimental Inoculation of Egyptian Fruit Bats (*Rousettus aegyptiacus*) with Ebola Virus. Viruses.

[8] Seifert SN (2020). *Rousettus aegyptiacus* bats do not support productive Nipah virus replication. J. Infect. Dis..

[9] Kulzer E (1963). Temperaturregulation bei Flughunden der Gattung Rousettus Gray. Z. Vergl. Physiol..

[10] Zhou P (2016). Contraction of the type I IFN locus and unusual constitutive expression of IFN-α in bats. Proc. Natl. Acad. Sci. U.S.A..

[11] Pavlovich SS (2018). The Egyptian Rousette genome reveals unexpected features of bat antiviral immunity. Cell.

[12] Subudhi S, Rapin N, Misra V (2019). Immune system modulation and viral persistence in bats: Understanding viral spillover. Viruses.

[13] Banerjee A, Rapin N, Bollinger T, Misra V (2017). Lack of inflammatory gene expression in bats: a unique role for a transcription repressor. Sci. Rep..

[14] Ahn M (2019). Dampened NLRP3-mediated inflammation in bats and implications for a special viral reservoir host. Nat. Microbiol..

[15] Guito JC (2021). Asymptomatic infection of Marburg virus reservoir bats is explained by a strategy of immunoprotective disease tolerance. Curr. Biol..

[16] Wang L-F, Gamage AM, Chan WOY, Hiller M, Teeling EC (2021). Decoding bat immunity: the need for a coordinated research approach. Nat. Rev. Immunol..

[17] Gamage AM (2020). Immunophenotyping monocytes, macrophages and granulocytes in the Pteropodid bat Eonycteris spelaea. Sci. Rep..

[18] Martínez Gómez JM (2016). Phenotypic and functional characterization of the major lymphocyte populations in the fruit-eating bat *Pteropus alecto*. Sci. Rep..

[19] Periasamy P (2019). Studies on B cells in the fruit-eating black flying fox (*Pteropus alecto*). Front. Immunol..

[20] Petriccione M, Mastrobuoni F, Zampella L, Scortichini M (2015). Reference gene selection for normalization of RT-qPCR gene expression data from *Actinidia deliciosa* leaves infected with *Pseudomonas syringae* pv. actinidiae. Sci. Rep..

[21] McMillan M, Pereg L (2014). Evaluation of reference genes for gene expression analysis using quantitative RT-PCR in *Azospirillum brasilense*. PLoS ONE.

[22] Radonić A (2004). Guideline to reference gene selection for quantitative real-time PCR. Biochem. Biophys. Res. Commun..

[23] Cruz-Neto AP, Garland T, Abe AS (2001). Diet, phylogeny, and basal metabolic rate in phyllostomid bats. Zoology (Jena).

[24] Hock RJ (1951). The metabolic rates and body temperatures of bats. Biol. Bull..

[25] O'Mara MT (2017). Cyclic bouts of extreme bradycardia counteract the high metabolism of frugivorous bats. Elife.

[26] Noll UG (1979). Body temperature, oxygen consumption, noradrenaline response and cardiovascular adaptations in the flying fox, *Rousettus aegyptiacus*. Comp. Biochem. Physiol. A Physiol..

[27] Brook CE (2020). Accelerated viral dynamics in bat cell lines, with implications for zoonotic emergence. Elife.

[28] Koh J (2019). ABCB1 protects bat cells from DNA damage induced by genotoxic compounds. Nat. Commun..

[29] Xie J (2018). Dampened STING-dependent interferon activation in bats. Cell Host Microbe.

[30] Bustin SA (2009). The MIQE guidelines: Minimum information for publication of quantitative real-time PCR experiments. Clin. Chem..

[31] La Cruz-Rivera PC, de,  (2018). The IFN response in bats displays distinctive IFN-stimulated gene expression kinetics with atypical RNASEL induction. J. Invest. Med..

[32] Klie M, Debener T (2011). Identification of superior reference genes for data normalisation of expression studies via quantitative PCR in hybrid roses (*Rosa hybrida*). BMC. Res. Notes.

[33] Pfaffl MW, Tichopad A, Prgomet C, Neuvians TP (2004). Determination of stable housekeeping genes, differentially regulated target genes and sample integrity: BestKeeper–Excel-based tool using pair-wise correlations. Biotechnol. Lett..

[34] Pombo MA, Zheng Y, Fei Z, Martin GB, Rosli HG (2017). Use of RNA-seq data to identify and validate RT-qPCR reference genes for studying the tomato-Pseudomonas pathosystem. Sci. Rep..

[35] Andersen CL, Jensen JL, Ørntoft TF (2004). Normalization of real-time quantitative reverse transcription-PCR data: a model-based variance estimation approach to identify genes suited for normalization, applied to bladder and colon cancer data sets. Can. Res..

[36] Dheda, K. *et al.* Validation of housekeeping genes for normalizing RNA expression in real-time PCR. *BioTechniques* **37,** 112–4, 116, 118–9. 10.2144/04371RR03 (2004). 10.2144/04371RR03 15283208

[37] Glare EM, Divjak M, Bailey MJ, Walters EH (2002). beta-Actin and GAPDH housekeeping gene expression in asthmatic airways is variable and not suitable for normalising mRNA levels. Thorax.

[38] Suzuki T, Higgins PJ, Crawford DR (2000). Control selection for RNA quantitation. Biotechniques.

[39] Falkenberg VR, Whistler T, Murray JR, Unger ER, Rajeevan MS (2011). Identification of Phosphoglycerate Kinase 1 (PGK1) as a reference gene for quantitative gene expression measurements in human blood RNA. BMC. Res. Notes.

[40] Molina CE (2018). Identification of optimal reference genes for transcriptomic analyses in normal and diseased human heart. Cardiovasc. Res..

[41] Sarwar MB (2020). Identification and validation of superior housekeeping gene(s) for qRT-PCR data normalization in Agave sisalana (a CAM-plant) under abiotic stresses. Physiol. Mol. Biol. Plants.

[42] Aminfar Z, Rabiei B, Tohidfar M, Mirjalili MH (2019). Selection and validation of reference genes for quantitative real-time PCR in *Rosmarinus officinalis* L. in various tissues and under elicitation. Biocatal. Agric. Biotechnol..

[43] Bai B, Ren J, Bai F, Hao L (2020). Selection and validation of reference genes for gene expression studies in Pseudomonas brassicacearum GS20 using real-time quantitative reverse transcription PCR. PLoS ONE.

[44] Ham S, Harrison C, Southwick G, Temple-Smith P (2016). Selection of internal control genes for analysis of gene expression in normal and diseased human dermal fibroblasts using quantitative real-time PCR. Exp. Dermatol..

[45] Huggett J, Dheda K, Bustin S, Zumla A (2005). Real-time RT-PCR normalization. Strategies and considerations. Genes Immun..

[46] Panina Y, Germond A, Masui S, Watanabe TM (2018). Validation of common housekeeping genes as reference for qPCR gene expression analysis during iPS reprogramming process. Sci. Rep..

[47] Sullivan-Gunn, M., Hinch, E., Vaughan, V. & Lewandowski, P. Choosing a stable housekeeping gene and protein is essential in generating valid gene and protein expression results. *Br. J. Cancer* **104,** 1055; author reply 1056. 10.1038/bjc.2011.35 (2011). 10.1038/bjc.2011.35 PMC3065275 21364583

[48] Fujii H (2010). Functional analysis of *Rousettus aegyptiacus* "signal transducer and activator of transcription 1" (STAT1). Dev. Comp. Immunol..

[49] Sabath DE, Broome H, Prystowsky MB (1990). Glyceraldehyde-3-phosphate dehydrogenase mRNA is a major interleukin 2-induced transcript in a cloned T-helper lymphocyte. Gene.

[50] Graven KK, McDonald RJ, Farber HW (1998). Hypoxic regulation of endothelial glyceraldehyde-3-phosphate dehydrogenase. Am. J. Physiol..

[51] Hazell AS, Desjardins P, Butterworth RF (1999). Increased expression of glyceraldehyde-3-phosphate dehydrogenase in cultured astrocytes following exposure to manganese. Neurochem. Int..

[52] Nakayama T (2018). Assessment of suitable reference genes for RT-qPCR studies in chronic rhinosinusitis. Sci. Rep..

[53] O'Neill LAJ, Kishton RJ, Rathmell J (2016). A guide to immunometabolism for immunologists. Nat. Rev. Immunol..

[54] O'Neill LAJ, Pearce EJ (2016). Immunometabolism governs dendritic cell and macrophage function. J. Exp. Med..

[55] Tannahill GM (2013). Succinate is an inflammatory signal that induces IL-1β through HIF-1α. Nature.

[56] Cham CM, Driessens G, O'Keefe JP, Gajewski TF (2008). Glucose deprivation inhibits multiple key gene expression events and effector functions in CD8+ T cells. Eur. J. Immunol..

[57] Cham CM, Gajewski TF (2005). Glucose availability regulates IFN-gamma production and p70S6 kinase activation in CD8+ effector T cells. J. Invest. Med..

[58] Chang C-H (2013). Posttranscriptional control of T cell effector function by aerobic glycolysis. Cell.

[59] Macintyre AN (2014). The glucose transporter Glut1 is selectively essential for CD4 T cell activation and effector function. Cell Metab..

[60] Gerriets VA (2015). Metabolic programming and PDHK1 control CD4+ T cell subsets and inflammation. J. Clin. Investig..

[61] Keating SE (2016). Metabolic reprogramming supports IFN-γ production by CD56bright NK cells. J. Immunol. (Baltimore, Md., 1950 ).

[62] Mah AY (2017). Glycolytic requirement for NK cell cytotoxicity and cytomegalovirus control. JCI Insight.

[63] Fekete T (2018). Human plasmacytoid and monocyte-derived dendritic cells display distinct metabolic profile upon RIG-I activation. Front. Immunol..

[64] Burke JD, Platanias LC, Fish EN (2014). Beta interferon regulation of glucose metabolism is PI3K/Akt dependent and important for antiviral activity against coxsackievirus B3. J. Virol..

[65] Pantel A (2014). Direct type I IFN but not MDA5/TLR3 activation of dendritic cells is required for maturation and metabolic shift to glycolysis after poly IC stimulation. PLoS Biol..

[66] Zhang W (2019). Lactate is a natural suppressor of RLR signaling by targeting MAVS. Cell.

[67] Kelm DH, Simon R, Kuhlow D, Voigt CC, Ristow M (2011). High activity enables life on a high-sugar diet: Blood glucose regulation in nectar-feeding bats. Proc. Biol. Sci..

[68] Amitai O (2010). Fruit bats (Pteropodidae) fuel their metabolism rapidly and directly with exogenous sugars. J. Exp. Biol..

[69] Tristan C, Shahani N, Sedlak TW, Sawa A (2011). The diverse functions of GAPDH: views from different subcellular compartments. Cell. Signal..

[70] Tarze A (2007). GAPDH, a novel regulator of the pro-apoptotic mitochondrial membrane permeabilization. Oncogene.

[71] Millet P, Vachharajani V, McPhail L, Yoza B, McCall CE (2016). GAPDH binding to TNF-α mRNA contributes to posttranscriptional repression in monocytes: A novel mechanism of communication between inflammation and metabolism. J. Invest. Med..

[72] Frick WF, Kingston T, Flanders J (2020). A review of the major threats and challenges to global bat conservation. Ann. N. Y. Acad. Sci..

[73] Gorbunova V, Seluanov A, Kennedy BK (2020). The world goes bats: Living longer and tolerating viruses. Cell Metab..

[74] Luis AD (2013). A comparison of bats and rodents as reservoirs of zoonotic viruses: are bats special?. Proc. Biol. Sci..

[75] Turmelle AS, Olival KJ (2009). Correlates of viral richness in bats (order Chiroptera). EcoHealth.

[76] Halwe NJ (2021). Egyptian fruit bats (*Rousettus aegyptiacus*) were resistant to experimental inoculation with avian-origin Influenza A virus of subtype H9N2, but are susceptible to experimental infection with bat-borne H9N2 virus. Viruses.

[77] Chomczynski P, Sacchi N (1987). Single-step method of RNA isolation by acid guanidinium thiocyanate-phenol-chloroform extraction. Anal. Biochem..

[78] Lee C, Kim J, Shin SG, Hwang S (2006). Absolute and relative QPCR quantification of plasmid copy number in Escherichia coli. J. Biotechnol..

