# Supplementary figures and images for: Selection and stability validation of reference gene candidates for transcriptional analysis in Rousettus aegyptiacus
Source: Sci Rep. 2021 Nov 4;11:21662. doi: 10.1038/s41598-021-01260-z (PMC8568961; doi:10.1038/s41598-021-01260-z)

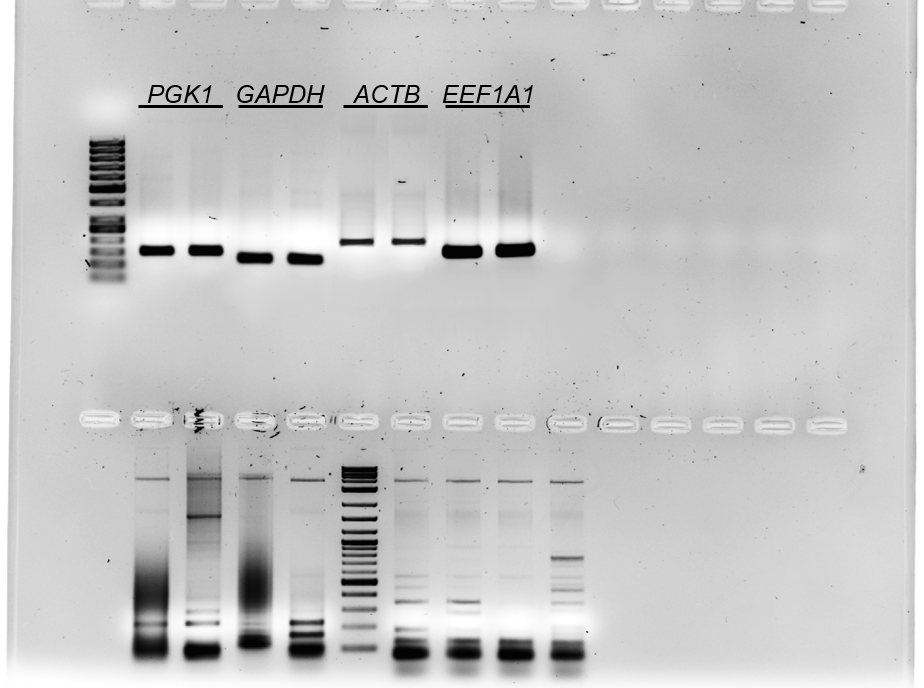

Supplement: Supplementary file 1 — Supplementary Information 1. [file 41598_2021_1260_MOESM1_ESM.tif]
