# Supplementary material for: Selection and stability validation of reference gene candidates for transcriptional analysis in Rousettus aegyptiacus
Source: Sci Rep. 2021 Nov 4;11:21662. doi: 10.1038/s41598-021-01260-z (PMC8568961; doi:10.1038/s41598-021-01260-z)
Supplement: Supplementary file 2 — Supplementary Information 2. [file 41598_2021_1260_MOESM2_ESM.docx]

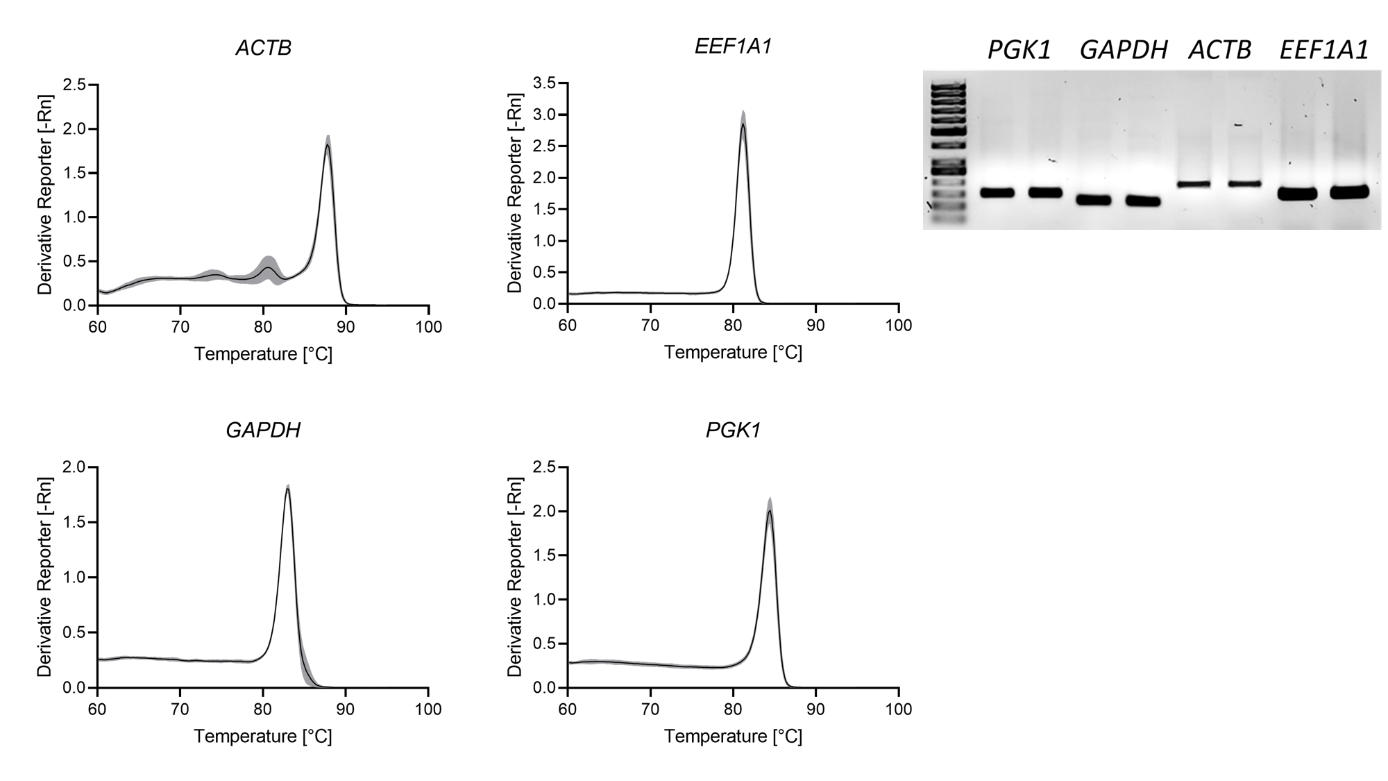
**Supplementary figures**

**Figure S1. Melting curve and gel electrophoresis analysis of primers for candidate reference genes** **of *Rousettus aegyptiacus*.** Melting curve analysis was conducted during qRT-PCR and amplicons were visualized in a 1.5% agarose gel.


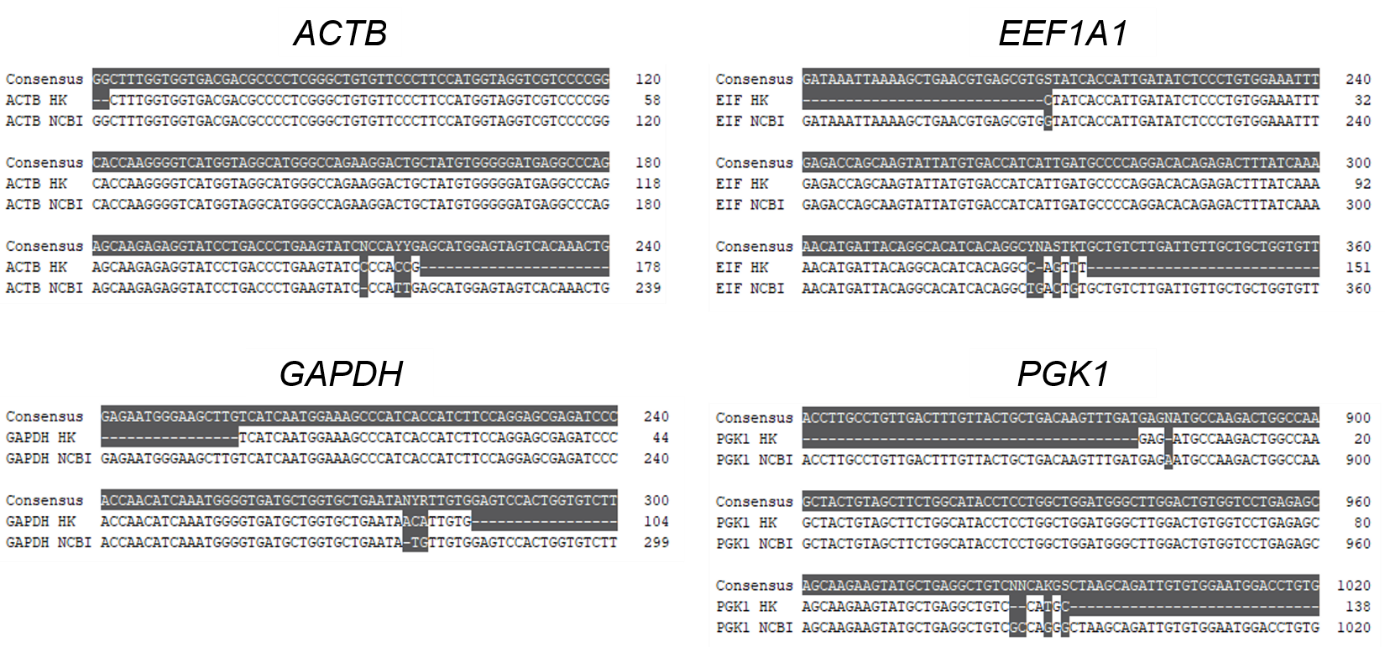


**Figure S2. Sequencing analysis of PCR amplicons for candidate reference genes of *Rousettus aegyptiacus*.** PCR amplicons were purified, sequenced and aligned with the NCBI reference genes to validate the specificity of primers.


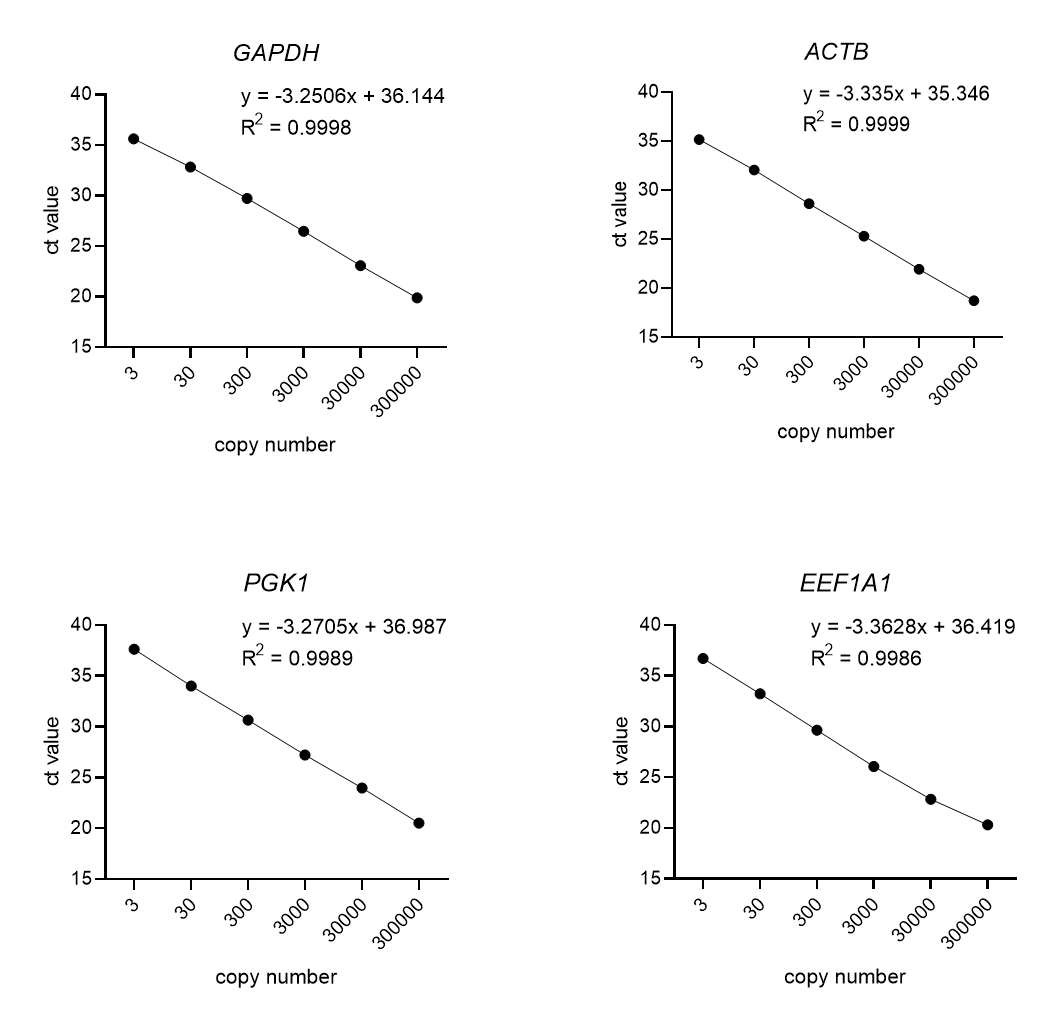


**Figure S3. Standard curves for candidate reference genes of *Rousettus aegyptiacus*.** Standard curves were generated with ranges from 3 to 300,000 copies and the slopes and correlation coefficient (R^2^) were determined.
